# How Multidisciplinary Are the Multidisciplinary Journals *Science* and *Nature*?

**DOI:** 10.1371/journal.pone.0152637

**Published:** 2016-04-04

**Authors:** Gregg E. A. Solomon, Stephen Carley, Alan L. Porter

**Affiliations:** 1 Division of Research on Learning, National Science Foundation, Arlington, Virginia, United States of America; 2 Department of Psychology, Harvard University, Cambridge, Massachusetts, United States of America; 3 School of Public Policy, Georgia Institute of Technology, Atlanta, Georgia, United States of America; 4 Technology Policy and Assessment Center, Georgia Institute of Technology, Atlanta, Georgia, United States of America; 5 Search Technology, Inc., Norcross, Georgia, United States of America; Universidad de Las Palmas de Gran Canaria, SPAIN

## Abstract

Interest in cross-disciplinary research knowledge interchange runs high. Review processes at funding agencies, such as the U.S. National Science Foundation, consider plans to disseminate research across disciplinary bounds. Publication in the leading multidisciplinary journals, *Nature* and *Science*, may signify the epitome of successful interdisciplinary integration of research knowledge and cross-disciplinary dissemination of findings. But how interdisciplinary are they? The journals are multidisciplinary, but do the individual articles themselves draw upon multiple fields of knowledge and does their influence span disciplines? This research compares articles in three fields (Cell Biology, Physical Chemistry, and Cognitive Science) published in a leading disciplinary journal in each field to those published in *Nature* and *Science*. We find comparable degrees of interdisciplinary integration and only modest differences in cross-disciplinary diffusion. That said, though the rate of out-of-field diffusion might be comparable, the sheer reach of *Nature* and *Science*, indicated by their potent Journal Impact Factors, means that the diffusion of knowledge therein can far exceed that of leading disciplinary journals in some fields (such as Physical Chemistry and Cognitive Science in our samples).

## Introduction

Interest in cross-disciplinary research knowledge interchange runs high. US funding agencies, including the National Science Foundation and the National Institutes of Health, as well as the National Academies of Science [1–4], promote programs with the explicit goal of fostering interdisciplinary research. And, over time, research in general is becoming more interdisciplinary [5].

Elsewhere we have explored interdisciplinary research counterposed against other forms of research, expressly disciplinary and multidisciplinary, with note of the potential of developing future fields via transdisciplinarity to go beyond previous fields [6–8]. Collectively, researchers in the field will sometimes bundle efforts extending beyond one discipline as cross-disciplinary. In this paper, we build on an operationally oriented definition from the National Academies report on *Facilitating Interdisciplinary Research*:

“Interdisciplinary research (IDR) is a mode of research by teams or individuals that integrates information, data, techniques, tools, perspectives, concepts, and/or theories from two or more disciplines or bodies of specialized knowledge to advance fundamental understanding or to solve problems whose solutions are beyond the scope of a single discipline or area of research practice” (p. 188) [1].

The essence here is the requirement of *integration* of knowledge (of various forms, potentially done by teams or individuals). A key distinction arises between *interdisciplinary*–as reflecting a greater degree of integration–and *multidisciplinary*–as more reflecting juxtaposition [6, 9].

In recognition of the promise that interdisciplinary research holds for addressing complex scientific problems with societal implications, the National Science Foundation (NSF) directs grant reviewers to consider a proposal’s plan to disseminate findings across disciplinary bounds in order to have a broader impact. One of us (GS), a program director at NSF, has frequently heard panelists reviewing grant proposals, program directors reviewing progress reports, and external evaluators reviewing the impact of funding programs comment as if publication in *Nature* or *Science* (in addition to affording a not inconsiderable “Stamp of Scientific Approval” for appearing in arguably the most important scientific journals in the world) signified in itself the successful interdisciplinary integration and diffusion of findings.

But does it? How interdisciplinary are *Nature* and *Science*? Articles in them are explicitly chosen not only because they move their specific literatures in important ways, but also because of their potential to interest–and to influence–multiple segments of the scientific community. To be sure, one has merely to pick up an issue at random to see authors from a wide range of scientific departments and institutions, questions pursued using a wide range of methods, and references drawn from a wide range of literatures. In our investigation, we recognize that *Nature* and *Science* are surely multidisciplinary, but here we explore the extent to which their content (their articles) are interdisciplinary.

What does multidisciplinarity at the level of the journals indicate about the integration and diffusion of knowledge at the level of individual articles? Researchers can appear to be promiscuously interdisciplinary, publishing with multiple collaborators in multiple fields, when at the individual project level they are serially monodisciplinary, each publication hewing closely to a particular literature. Similarly, the goal of many funding programs is the creation of a multidisciplinary portfolio of research projects. When evaluated at the level of the portfolio, they appear successfully interdisciplinary, but when evaluated at the level of individual funded projects they can look decidedly monodisciplinary [10]. Level of analysis is critical even in an exploratory evaluation, an important lesson to keep in mind in this time of data-driven accountability [11–12].

Our overarching research questions concern patterns of research knowledge transfer across fields, motivated by the hypothesis that interdisciplinary research offers compelling promise in advancing science and solving complex societal problems. We translate these interests to examine empirical evidence on citation patterns across scientific fields, comparing *Nature* and *Science* articles to those appearing in prominent disciplinary journals in those selected fields. Our focal questions: Are *Nature* and *Science* papers more interdisciplinary? And do *Nature* and *Science* papers diffuse strikingly more widely?

## Data and Methods

### Defining the fields, selecting the journals, and acquiring the dataset

We chose *Nature* and *Science* because they are the two leading multidisciplinary journals–based on reputation, explicit editorial policy, Web of Science Subject Category (i.e., MULTIDISCIPLINARY SCIENCE), and prestige–fortified by their very high Journal Impact Factors (JIF). Though JIF tells a limited story [13–14], *Nature’s* and *Science’s* high JIFs do indicate that their articles are read and cited. A lot. Using JIFs from the 2010 Journal Citation Reports, we find *Nature* at JIF = 36.1 and *Science* at JIF = 31.4. We utilize Web of Science (WoS) as a leading database indexing fundamental research published in over 12,000 journals.

Data challenges led us to limit our exploration to select fields, mainly because collection and cleaning of sets of citing records is extremely expensive, both in terms of time and funding. Articles typically cite 30 or more references, resulting in a huge multiplier. Furthermore, articles in the top multidisciplinary journals–of special interest to us–accrue many citations. For example, one compilation of 545 publications we examined drew 60,425 cites.

In order to address questions of the interdisciplinarity of *Nature* and *Science*, we focused on a sample of articles that appeared in a single year, 2009, in each of three fields–a life science (Cell Biology), a physical science (Physical Chemistry), and a behavioral science (Cognitive Science). These choices resulted from discussions with NSF colleagues about candidate fields in terms of coherence and mainstream interest, and empirical forays regarding journal coverage. We observed that any given issue of *Nature* or *Science* might have multiple articles on Cell Biology, whereas it might have none on Physical Chemistry or Cognitive Science.

We then sought to identify a comparison set of top disciplinary journals. As a starting place, we used the Web of Science Subject Categories (WCs) to identify the set of journals in each field covered by WoS. We then considered journals with relatively high JIFs for their fields (one indicator of impact), looked to whether they were the flagship journals of the major professional societies in the fields, and again consulted with NSF colleagues on prestige and reputation. We selected the following:

- *Cell* (JIF = 32.4)
- *Journal of Physical Chemistry A* (JIF = 2.8)
- *Cognitive Science* (JIF = 2.3)

We recognize that the wide range in impact of the journals affects the relative diffusion of research knowledge across disciplines. As the above JIFs attest, citation practices vary greatly by field [15]. JIF influence seems to peak after about three years [16]. Other variables influencing citation include how basic or applied the science is (basic research attracts more citations [17]), average number of authors per article (more authors attract more citations [18]), and average number of affiliations per article (more affiliations attract more citations [18]). Fields’ publishing norms vary in terms of co-authoring, author ordering, average number of cited references, self-citing, and so on. The message is to be cautious in comparing fields in analyses such as this one [19]. Hence, we key on within-field comparisons of *Nature* and *Science* articles to disciplinary articles and refrain from field-to-field comparisons.

Number of publications to assess was another consideration. Obtaining sufficient publications factored into our field, journal, and temporal (one year) selections. We decided that looking at publications appearing in 2009 would support conclusions that are reasonably current, yet allow sufficient time (5 years) for papers to accrue citations; we note that there are many issues in tracking citation over time [20]. We followed up by collecting WoS records that have cited the 2009 publications through 2014. We used the WoS “document type” information to restrict our search to “articles” (setting aside reviews and various editorial content publications). Then we considered how to sample articles, deciding against going after the most cited papers as this might distort our assessment of knowledge diffusion patterns and differences. We moved toward a set of criteria that balanced sizes for samples from *Nature* and *Science* and the disciplinary journals, while taking best advantage of the articles available for 2009. We did not include in our sample articles that had fewer than four references to WoS journals as that would have rendered the interdisciplinarity metrics unreliable. Using these criteria, we gathered a sample of 1643 *Nature* and *Science* articles published in 2009.

#### Determining what articles are in which fields

A key challenge was to determine what constitutes an article in a target field. For the three disciplinary journals, we took publication in the chosen journal as the selection criterion–that is, an article appearing in *Cognitive Science* is considered by definition to be a Cognitive Science article. But how to categorize *Nature* and *Science* articles? We considered various ways to classify articles, including assignment based on review of titles, keywords, or abstracts by disciplinary experts. The greater subjectivity of such judgments, combined with the sheer numbers of papers to be examined, rendered the approach less attractive (though not inappropriate). We also considered coding authors’ disciplines based on departmental affiliation. That would have proven problematic. First, it is not clear who the lead author of an article is. In some fields, the first author can be assumed to be the lead, in others it is the last. Moreover, *Nature* and *Science* do not always show departmental affiliation. And even when they do, the specificity of our fields does not always satisfactorily match the available information–“Physical Chemistry” would not usually be the name of a department or center. Furthermore, affiliation information gleaned from WoS records is inconsistent and deciding on field boundaries is tricky. Further ambiguity arises with multiple authors who reside in different departments or even individual authors with multiple departmental affiliations.

We shifted to examination of a *Nature* or *Science* article’s cited references as a viable, reproducible, and somewhat more objective indicator of field ties. We follow Schunn [21] whose commonsense reasoning held that if 25% or more of the citations by a paper were to a given field, then that field should be considered to be a major influence. We therefore search the references of the articles appearing in *Nature* and *Science* and include all of those for which 25% of the references are in a particular field. We recognize that this approach tiptoes toward tautology, but we reasoned that if, for example, at least 25% of an article’s references were not to articles appearing in Cell Biology journals, it would be difficult to consider the article to be a Cell Biology article. As a further check, we sampled entire issues of *Nature* and *Science* from our dataset and found that for every article there was at least one field that accounted for at least 25% of its references. This is not to say this need be true for the entire dataset, but our inclusion criterion would not appear to have biased our dataset in a systematic fashion.

To perform the categorization, we apply a thesaurus in *VantagePoint* software [22] to standardize journal names, then another to associate cited journals to their WCs (thesaurus provided by Thomson Reuters). Operationalizing *Nature* and *Science* article selection requires consideration of multiple factors. Reflecting on our selection of “disciplines” to study regarding multidisciplinary knowledge transfer patterns, we are using WCs. The names suggest the tendency of fields of study to intersect and integrate over time. Again, in consultation with disciplinary experts and scope provided by professional societies, we include the following WCs for our fields:

- **Cell Biology**: CELL BIOLOGY; BIOCHEMISTRY & MOLECULAR BIOLOGY
- **Physical Chemistry**: CHEMISTRY, PHYSICAL; PHYSCIS, ATOMIC, MOLECULAR & CHEMICAL
- **Cognitive Science**: BEHAVIORAL SCIENCES; COMPUTER SCIENCE, ARTIFICIAL INTELLIGENCE; LINGUISTICS; PSYCHOLOGY; PSYCHOLOGY, APPLIED; PSYCHOLOGY, DEVELOPMENTAL; PSYCHOLOGY, EDUCATIONAL; PSYCHOLOGY, EXPERIMENTAL; PSYCHOLOGY, MATHEMATICAL; PSYCHOLOGY, MULTIDISCIPLINARY; PSYCHOLOGY, SOCIAL

#### Resulting dataset

Of the 1643 articles appearing in *Nature* and *Science* in 2009, our search, using the WC-based operationalization described above, yielded 339 Cell Biology articles, 61 Physical Chemistry articles, and 34 Cognitive Science articles. All of the selected *Nature* and *Science* articles are in only one of the three field categories; that is, there is no overlap. Preliminary analyses on the metrics described below showed no significant differences between articles in a given field appearing in *Nature* as opposed to those appearing in *Science*. Therefore, because the numbers of articles in Physical Chemistry and Cognitive Science appearing in *Nature* and *Science* were relatively low, and those in Cell Biology so high, the articles appearing in the two journals were pooled. As the pooled number of Cell Biology articles was so great, we randomly selected 50 articles from *Science* and 50 from *Nature*. Finally, we randomly sampled 50 articles appearing in 2009 from each of the three disciplinary journals (*Cell*, *Journal of Physical Chemistry–A*, and *Cognitive Science*) for inclusion in our dataset as the foil to the *Nature* and *Science* articles.

### Metrics of interdisciplinarity

#### Integration and Diffusion scores

Measuring interdisciplinarity remains a challenge [23]. Integration and Diffusion scores provide the foundational metrics for the present analyses. They were devised by program evaluators in support of the US National Academies Keck Futures Initiative [4] to help assess the interdisciplinarity of bodies of publication–for example, of a field, a department or center, or even compilations at a national level [6–8]. Integration scores are based on the diversity of a paper’s cited references [6, 24], with diversity operationalized using WCs. As accessed through Thomson Reuters Web of Knowledge site for 2014 publications, WoS distinguishes about 224 WCs in the natural sciences (Science Citation Index) and social sciences (Social Sciences Citation Index).

Integration scores reflect distance among WCs based on the relative degree to which one WC’s journals cite those of the other 223 WCs in a given year of WoS [25–26]. For example, WCs such as Ornithology and Forestry, whose journals frequently cite each other, are more similar than are, say, Acoustics and Economics, whose journals do not. Integration scores range from 0 toward 1, increasing as a paper’s references span more WCs, are distributed more evenly across those WCs, and are more distant from each other. WCs are assigned at the journal level [8], so they don’t reflect article content directly. Integration scores equate to Rao-Stirling diversity [24, 26] as a tri-dimensional concept operationalized in terms of: (i) variety (number of WCs cited), (ii) balance (evenness of distribution of citing those WCs), and (iii) disparity (dissimilarity of the cited WCs). For some purposes, separating those three aspects may be fruitful [27–28], but we combine them into a single indicator of degree of interdisciplinarity here (i.e., Integration scores).

Integration scores can be expressed as:

$$I=1-\sum_{i,j}s_{y}p_{i}p_{j}$$

where *p*_*i*_ is the proportion of references citing the WC *i* in a given paper. The summation is taken over the cells of the WC x WC matrix. *s*_*ij*_ is the cosine measure of similarity between WCs *i* and *j*—based on cross-citation among WoS journals in a given year (we use 2010 here). We report findings in terms of means, though we recognize that citation distributions are generally non-normal. For reporting purposes, use of means appears reasonable in that our focus is on scores that are constrained to a range from 0 to 1. That said, the analyses below were conducted using nonparametric statistics. As noted, WCs whose journals frequently cite each will be scored as more similar. If a given article cites a number of references from an array of academic disciplines distantly related it is said to be relatively *integrative*.

Diffusion scores can be thought of as Integration in reverse. The formulation is the same, except using the set of papers referencing the given paper to obtain the *citing* WCs. While Integration scores are backward looking in that they measure diversity among *cited* references, Diffusion scores are forward looking in that they measure diversity among papers that refer to a given body of work. Scholarship cited by a broad array of distantly related academic disciplines is said to be relatively *diffuse* [15].

Given that citations for a given article can accrue ad infinitum into the future, Diffusion scores can change over time. Hence, we want to compare papers of approximately the same age. Carley and Porter [15] provide benchmark Integration and Diffusion scores for WoS publications published in 1995, allowing an extended post-publication period in which to be cited. We note that suitable periods vary by field. For example, one would expect History citations to average much longer lags than, say, Nanotechnology. Here we calculate Diffusion scores for 2009 publications over an approximately 5-year period, through 2014, a reasonable period for citations to accrue [20].

#### In-field and out-of-field citations

We also use the WCs in a somewhat rougher fashion to determine whether a citing paper is out-of-field or in-field. If the citing article’s journal is categorized into any of the WCs for one of our three fields of interest, we considered it to be an in-field citation, even if the journal is also categorized in some other WC. (Relatively few journals appear in more than two WCs, but multiple categories for a given journal pose analytical challenges [29].) For example, if a target Cognitive Science article were cited by a paper appearing in a journal that is included in the WC “PSYCHOLOGY, DEVELOPMENTAL,” then the citation would be considered in-field. We tabulate the mean and median numbers of total citations for the articles in each field as well as the mean and median numbers of citations appearing in out-of-field journals.

To be sure, cross-disciplinarity is but one dimension of knowledge creation and diffusion; one could certainly consider others. And measures of cross-disciplinarity could use units other than WCs, such as departmental affiliations or final academic degrees of co-authors [30]. But WCs are highly suitable for study of cross-disciplinarity in several regards. First, their granularity corresponds well to that advanced by the National Academies Committee on Facilitating Interdisciplinary Research [1]. Second, WCs are widely used by the bibliometrics community, allowing for a greater basis of comparison. Third, WCs are readily accessible from WoS abstract records. And finally, though exact assignment of journals to WCs can be problematic [31], miscategorizations should exert relatively modest effects on similarity measures as “correct” categorizations tend to be nearby [32].

## Results

**Fig 1:**
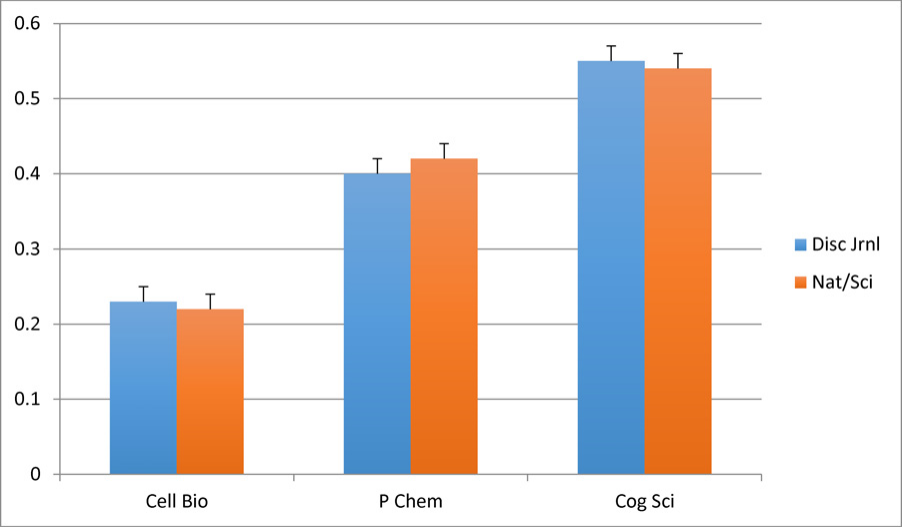
Mean Integration Scores of Articles Appearing in Disciplinary Journals Compared to those in *Nature/Science*, by Field. Cell Bio = Cell Biology, P Chem = Physical Chemistry, Cog Sci = Cognitive Science, Disc Jrnl = Disciplinary journal, Sci/Nat = *Science* and *Nature*.

The mean Integration scores for the journals in each field can be seen in Fig 1. Differences among the three fields under study are substantial. Carley and Porter [15] reported benchmark Integration and Diffusion scores for a set of disciplines based on all articles in those fields indexed by Web of Science in 1995. Consistent with those findings, we find differences between fields in the current study in the diversity of articles cited, even within *Nature* and *Science*. The mean Integration score of 0.22 for the Cell Biology articles in our study is highly disciplinary, approaching the lowest score derived in the benchmark study, 0.21 for “Mathematics.” The mean of 0.42 for *Nature* and *Science* articles in Physical Chemistry is similar to the relatively interdisciplinary score of 0.40 derived in the benchmark study for “Atomic, Molecular, and Chemical Physics,” and the mean of 0.54 for the Cognitive Science articles exceeds the highest Integration score in the benchmark study, the 0.43 derived for the related field of “Neuroscience.”

Critically, the articles in *Nature* and *Science* within each field are not significantly more interdisciplinary than are those sampled in the disciplinary journals, as indicated by preplanned Mann-Whitney *U* tests comparing the Integration Scores of the references in our sample. As can be seen more qualitatively in Table 1, the top five journals cited by the *Nature* and *Science* articles are not terribly different from those cited by the disciplinary journals within each field.

**Table 1:** Top Five Journals Cited by or Citing Target Articles, by Field and Journal Type.

	Cited Journals		Citing Journals	
Field	By Disciplinary Journal Articles	By *Nature/Science* articles	By Disciplinary Journal Articles	By *Nature/Science* articles
**Cell Biology**	*Cell; PNAS; Nature; Science; Embro*	*Cell; Nature; Science; PNAS; J Biol Chem; Mol Cell*	*PLOS ONE; PNAS; Cell; J Biol Chem; Nature*	*PLOS ONE; PNAS; J Biol Chem; Cell; Nature*
**Physical Chemistry**	*J Chem Physics; J Physical Chem–A; JACS; Chem Physics Letters; J Physical Chem—US*	*Science; Nature; Physics Rev Lett; JACS; J Chem Physics*	*J Physical Chem–A; Phys Chem Chem Phys; J Chem Physics; Chem Physics Letters; JACS*	*Phys Chem Chem Phys; J Phys Chem Lett; JACS; Physics Rev Lett; Science*
**Cognitive Science**	*Cognition; Psych Review; Science; Cognitive Psych; Psych Science*	*Science; Nature; PNAS; Psych Science; Nature Neuro*	*Cognitive Science; Cognition; Frontiers in Psych; PLOS ONE; JEP-General*	*PLOS ONE; PNAS; Neuroimage; J of Neuroscience; Science*

Even if papers appearing in *Nature* and *Science* are not generally more interdisciplinary than are those appearing in top disciplinary journals, as measured by their cited references (putative *influences* on the research conducted), they might yet be shown to be more multidisciplinary in the diversity of the papers citing them (putative *impact*). Given the great number of eyeballs scanning the pages of *Nature* and *Science* and the disciplinary diversity of their owners, the question at hand is whether gaze translates into influence. In order to answer this question, we next examine the articles that cite the sampled articles.

**Fig 2:**
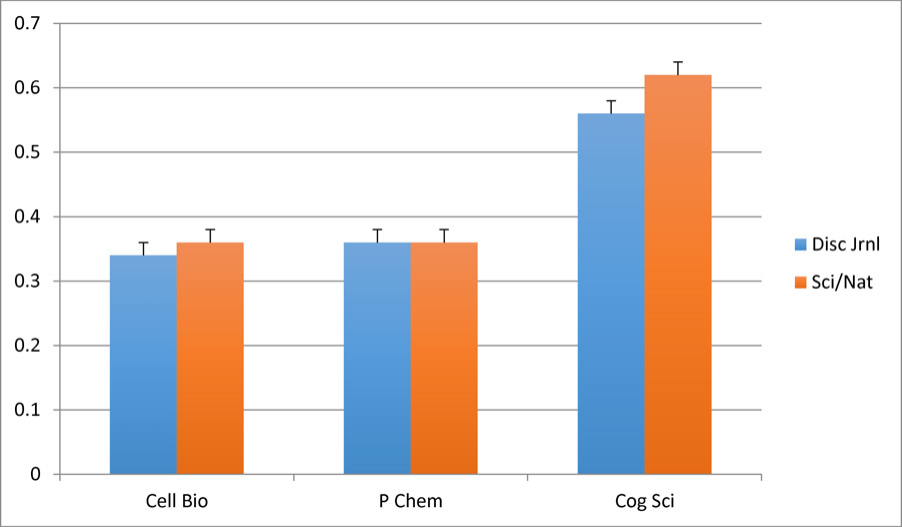
Mean Diffusion Scores of Articles Appearing in Disciplinary Journals Compared to those in *Nature/Science*, by Field. Cell Bio = Cell Biology, P Chem = Physical Chemistry, Cog Sci = Cognitive Science, Disc Jrnl = Disciplinary journal, Sci/Nat = *Science* and *Nature*.

Fig 2 shows the mean Diffusion scores for the original target journals, reflecting the diversity of journals in which those citing articles appeared. Preplanned Mann-Whitney *U* tests reveal no significant mean differences between Cell Biology articles appearing in *Cell* and those appearing in *Nature* and *Science*, nor for the Physical Chemistry articles appearing in the *Journal of Physical Chemistry—A* versus those appearing in *Nature* and *Science*. As was the case when looking at the journals *cited by* our target articles, the top five journals *citing* the target *Nature* and *Science* articles in these fields are not qualitatively different from those of the disciplinary journals (see Table 1). The story is slightly different for Cognitive Science. The mean Diffusion score of the target articles appearing in *Nature* and *Science* is higher than that for *Cognitive Science*, reaching significance in a one-tailed Mann-Whitney *U* test (*p* = .006). Significant, but the relatively small differences between means is hardly what one would have expected given the reputations of *Nature* and *Science* for multidisciplinary reach.

Before we dismiss the idea of there being a substantial cross-disciplinary knowledge transfer advantage for articles appearing in *Nature* and *Science*, let us consider raw citation counts. Do articles appearing in *Nature* and *Science* garner more citations out-of-field? As can be seen in Table 2, the mean total citations of Cell Biology articles appearing in *Nature* and *Science* is about the same as the total citations of articles appearing in *Cell*. This makes sense, given their similar JIFs. More to the point, the mean number of citations in out-of-field journals is also about the same.

**Table 2:** Mean number of total citations per article and mean number of citations per article appearing in journals that are out-of-field, by field.

Field	Journal	Mean Cites per Article	Mean Cites Out-of-Field per Article
Cell Biology	*Nature-Science*	165	86
Cell Biology	*Cell*	164	84
Physical Chemistry	*Nature-Science*	291	175
Physical Chemistry	*J Phys Chem–A*	12	6
Cognitive Science	*Nature-Science*	95	37
Cognitive Science	*Cognitive Science*	14	3

By contrast, articles appearing in the disciplinary Physical Chemistry and Cognitive Science journals receive dramatically fewer mean citations per article than do articles in those fields appearing in *Nature* and *Science* (Table 2). Similarly, the mean numbers of out-of-field citations are also dramatically lower. Unlike *Cell*, in the field of Cell Biology, no journal in Physical Chemistry or Cognitive Science has anything like the JIF of *Nature* and *Science*. As it turns out, sometimes JIF matters.

## Discussion and Conclusion

Our exploratory paper has produced findings that are at once both consistent and at odds with the reputation of *Nature* and *Science*. Integration scores–a measure of the diversity of the cited references–for the Cell Biology, Physical Chemistry, and Cognitive Science articles appearing in *Nature* and *Science* were not significantly greater than were those appearing in leading disciplinary journals in each field. Similarly, the Diffusion scores–a measure of the diversity of the citing references–for the Cell Biology and Physical Chemistry articles were not significantly different from those for articles appearing in leading disciplinary journals, and the Diffusion scores for the Cognitive Science articles, while statistically different, do not suggest a substantial practical difference.

The lack of substantial differences in the rate of multidisciplinary diffusion notwithstanding, the raw impact of *Nature* and *Science* remains. Articles appearing in *Cell* (whose JIF is comparable to that of *Nature* and *Science*) are cited as often as are those appearing in *Nature* and *Science*, and they are cited as often out of field. But articles appearing in *Nature* and *Science* are cited far more often than are articles in such top disciplinary journals as the *Journal of Physical Chemistry–A* and *Cognitive Science*, and so the articles in them are cited more times out of field.

It is worth considering several methodological and analytic decisions we made in this study. We contrasted Cell Biology, a life science with relatively high numbers of articles appearing in *Nature* and *Science*, with Physical Chemistry and Cognitive Science, represented by relatively low numbers of articles. Indeed, articles in the latter two fields appear so infrequently that one might question how representative of the field they are. Certainly *Nature* and *Science* could not be said to be journals of record in those fields. It is an empirical question whether these results might hold for other disciplinary journals within those fields. For example, we chose what we thought would be more focused contrast cases. We might instead have chosen to look at journals that reach across subfields within each discipline, such as the *Journal of the American Chemistry Society*, *Psychological Science*, or *PLOS Biology*. We might also have chosen other prominent multidisciplinary journals, such as *PLOS ONE*, *PNAS*, or *Journal of the Royal Society Interface*. And it is an empirical question what an examination of other disciplines would reveal.

Another important methodological decision in work of this type is choice of measures. As we noted above, there is no consensus on indicators of interdisciplinarity. We chose to use Integration and Diffusion scores, based on the Rao-Stirling diversity index, because of the appeal of its underlying conceptual basis (with its emphasis on disparity, balance, and variety), its standing in the scientometric community, and the existence of published benchmarks against which we could compare our results. But a case could certainly be made for using other measures. Zhang, Rousseau, and Glanzel [33], for example, argue for using their D2S measure that emphasizes variety over disparity and balance as an indicator of interdisciplinary diversity, and Bergmann and his colleagues [34] argue for quantifying interdisciplinarity by means of Jenson-Shannon divergence. Nonetheless, as suggested by our admittedly rough calculation of citation out of field and our qualitative comparison of the top cited and citing journals, the differences in integration and diffusion between the articles in *Nature* and *Science* and those in the disciplinary journals were not great. It is beyond the scope of this paper to address concerns about which measure of interdiscipinarity is best at addressing more subtle questions, such as what are optimal levels of interdisciplinarity in citations or in team composition. Suffice it to say that we were not looking for subtle effects.

We further acknowledge that bibliometric analyses themselves provide an imperfect perspective, especially for evaluative purposes, as Lane and others note, [13–14, 17, 35]. We might have chosen “Altmetrics” in order to tap into wider communities’ referencing (e.g., Google Scholar) or topical attention (e.g., Twitter activity) in order to add dimensions beyond WoS citation analyses (c.f., [36]). Such data would offer interesting resources to expand our inquiry into the ramifications of publication in *Nature* and *Science* through the attention drawn by various publics quite distinct from citation by formal research communities [37–39]. And, of course, there are other ways to operationalize interdisciplinarity, including text analyses of content similarity and research network span [40]. We also recognize other forms of diversity pertinent to gauging the merits of publication in multidisciplinary, as opposed to disciplinary journals. For example, do the former generate more awareness (e.g., reading) and utilization (e.g., as indicated by citation) across geographical entities (a candidate indicator of breakthrough potential along with interdisciplinarity [41])?

Clearly, our paper is meant to be less of a definitive work and more of an exploration of how one might go about addressing such issues of interdisciplinarity and multidisciplinarity. And to that end our findings certainly demonstrate that level of analysis matters. As always in science, one must take care to square the kinds of claims one would like to make with the kinds of evidence produced. In this case, the extreme diversity of the articles appearing in any given issue of *Nature* or *Science* was not matched by a similar diversity at the level of the individual article.

How interdisciplinary *should* the articles in Nature and Science be? We could not say. Upon reflection, our finding of the lack of substantially increased interdisciplinarity in the articles is actually quite reasonable, given the missions and editorial policies of the journals. Articles are chosen for publication in *Nature* and *Science* because, foremost, they are the products of excellent science. The history of science suggests that paradigm-busting work, as it were, is most productive when the science demands it [42]. Interdisciplinarity is not a “scientific good” in and of itself, nor is gratuitous multidisciplinary citation a necessary indicator of good science.

Does publication in *Nature* and *Science* signify the successful multidisciplinary diffusion of findings? Well, yes, to some degree. The articles appearing in them are not inherently of greater interest to “outsiders” as indicated by measures of the diversity of citing articles, but, in keeping with differences in JIF, they can attract a markedly greater number of out-of-field citations, likely deriving in part from their vetted quality and high profile. But even this claim must be qualified, for it varies by field. Thus, the moral of our story, at least for Physical Chemists and Cognitive Scientists seeking a broader impact: Do excellent work and publish it in *Nature* or *Science*.
